# The Case of Ketamine Allergy

**DOI:** 10.5811/cpcem.2017.7.34405

**Published:** 2017-10-03

**Authors:** William Bylund, Liam Delahanty, Maxwell Cooper

**Affiliations:** Department of Emergency Medicine, Naval Medical Center Portsmouth, Portsmouth, Virginia

## Abstract

Ketamine is often used for pediatric procedural sedation due to low rates of complications, with allergic reactions being rare. Immediately following intramuscular (IM) ketamine administration, a three-year-old female rapidly developed facial edema and diffuse urticarial rash, with associated wheezing and oxygen desaturation. Symptoms resolved following treatment with epinephrine, dexamethasone and diphenhydramine. This case presents a clinical reaction to ketamine consistent with anaphylaxis due to histamine release, but it is uncertain whether this was immunoglobulin E mediated. This is the only case reported to date of allergic reaction to IM ketamine, without co-administration of other agents.

## INTRODUCTION

Ketamine is a common medication, used in isolation as well as with other agents, for pediatric sedation in the emergency department (ED). It is often turned to because of its efficacy, ease of use, and favorable safety profile. Common side effects of ketamine when given intravenously or intramuscularly include over-sedation, increased oral secretions, tachycardia, vomiting and laryngospasm.^1^ The following is a case of an apparent anaphylactic reaction to a single dose of intramuscular (IM) ketamine for pediatric procedural sedation.

## CASE REPORT

A three-year-old female, without significant past medical history, presented to the ED with a one-centimeter linear laceration through the right lower lip secondary to collision with a domestic dog. The laceration crossed the vermillion border but did not penetrate the buccal mucosa, and no other injuries were noted. Due to the location of the laceration and the desire for good cosmesis, a decision was made to repair the laceration under sedation with IM ketamine.

A pre-sedation history and physical exam was performed. The patient’s mother stated that the patient had a history of seasonal allergies and asthma triggered by environmental allergens, but had received no allergy or over-the-counter pain medications the day of presentation. The pre-sedation airway exam was unremarkable. The patient was attached to continuous cardiac monitor, end tidal CO2 (ETCO2) and pulse oximetry (POx), in addition to being placed on two liters nasal cannula (NC). No intravenous (IV) access was obtained prior to sedation. Seventy milligrams (4.4mg/kg) of ketamine was administered IM into the right thigh.

Within two minutes of administration, the patient developed facial edema and diffuse urticarial rash to face and torso. IV access was immediately obtained. Spontaneous breathing continued, but audible expiratory wheezing was noted. Auscultation of the patient’s lungs revealed diffuse wheezing. An immediate request was made for IM epinephrine and a bag valve mask (BVM) was placed over the NC. An associated POx decrease to the 80s was noted but improved quickly with BVM. Copious oral secretions were noted, requiring aggressive suctioning. The patient’s airway was repositioned into a sniffing position with folded blankets and 0.15mg of IM epinephrine was given approximately 15 minutes after initial ketamine. This was followed by 8mg dexamethasone IV and 25mg IV diphenhydramine. The patient’s wheezing and urticarial rash improved, and her lip laceration was repaired in the standard fashion. The patient emerged from the sedation approximately one hour after administration of ketamine. She recovered completely within the following 30 minutes and was monitored for an additional three hours prior to discharge. Of note, the time estimates above are based on the average retrospective recall of events. No staffing was available for real-time charting.

## DISCUSSION

Ketamine is commonly used for procedural sedation and analgesia in the ED. Physicians often prefer the agent because adverse effects are very rare.^1^ The incidence of apnea is less than 0.1%. Aspiration, hypotension, and bradycardia similarly occur at low rates.^1^ One particularly feared adverse effect is laryngospasm, an involuntary titanic contraction of the vocal cords. It is characterized by stridor, and usually resolves spontaneously in less than a minute. However, it is a potentially life-threatening condition with an incidence of 0.5% secondary to ketamine sedation.^1^

Our patient’s symptomatology was consistent with activation of mast cells and release of preformed mediators (e.g., histamine), though we cannot be certain whether this event was immunoglobulin E (IgE) mediated. Previously, the term anaphylaxis was used to identify the IgE dependent pathway and anaphylactoid the IgE independent pathway. Both pathways lead to degranulation of basophils and mast cells with release of preformed mediators. The term anaphylaxis is now defined as a life-threatening allergic reaction occurring rapidly after exposure, and involves two or more organ systems.^2^ The rapid onset facial edema, urticarial rash, and diffuse pulmonary wheezing in our case is not typical of laryngospasm. A meta-analysis by Bellolio et al. revealed no cases of anaphylaxis in 13,883 pediatric sedations, most of whom received ketamine. Adverse drug reactions consistent with anaphylaxis induced by ketamine are rare events, and when present do not appear to be IgE mediated. Thus, it is impossible to predict future reactions.^1^

We performed a literature search for both adverse drug reactions and allergic reactions to ketamine, and discovered six case reports.^3^–^8^ In all but one case, ketamine was used for pediatric procedural sedation or general anesthesia. Ozcan et al. described a true type I hypersensitivity reaction to IV ketamine and midazolam infusion, manifested by pruritic urticarial rash and perioral edema. The sensitivity reaction was confirmed by elevated tryptase level taken two hours after the event, as well as intradermal skin testing afterwards. Nguyen et al. reported an allergic reaction to IV push ketamine, which followed administration of fentanyl and ondansetron. Diffuse morbilliform rash resolved within five minutes after administration of diphenhydramine.

Karayan et al. reported a generalized rash and laryngospasm following ketamine administration, though the full report was not available in English. Nwasor et al. reported an allergic reaction following administration of IM ketamine and IV atropine: urticarial rash, difficulty breathing and subsequent hypoxia to 90% by POx. Endotracheal intubation was performed, and symptoms resolved with IV hydrocortisone. Matheieu et al. report a case of extensive macular rash after ketamine and hyoscine were given IM. Boynes reported an allergic reaction with severe urticarial rash and wheezing similar to our case report, following IM ketamine and midazolam prior to a dental procedure. In their case, intubation was performed prophylactically.

**CPC-EM Capsule**

What do we already know about this clinical entity?

Ketamine is commonly used for procedural sedation. Allergic reactions are rare, and anaphylaxis is only reported in combination with other drugs.

What makes this presentation of disease reportable?

This is the first reported case of anaphylaxis definitively associated with ketamine, as it was the only drug administered.

What is the major learning point?

Emergency physicians (EP) should have increased awareness of rare allergic reactions, with better preparedness for them.

How might this improve emergency medicine practice?

EPs will better anticipate anaphylaxis during procedural sedation, and subsequently treat more rapidly.

Although these cases are similar, all of them involve co-administration of other agents. Our case is the only adverse drug reaction reported thus far in the setting of IM ketamine as a monotherapy. We performed an additional literature search for adverse drug reaction to benzethonium chloride, the preservative used in our ketamine supply. No studies were found to discuss this as a possible alternative cause of allergic reaction. Latex exposure was considered, but patient had no prior latex allergy, and patient had no known latex exposure prior to her reaction.

Adverse drug reactions are often immune-mediated hypersensitivity reactions, as opposed to anaphylactic reactions, which are mediated by IgE and classified as type I allergic reactions.^5^,^8^ The clinical criteria for anaphylaxis generally include urticaria and one of the following: respiratory distress, hypoxia, hypotension, or associated symptoms of organ dysfunction. Symptoms occur within minutes to hours after allergen exposure. Activation of mast cells and basophils from IgE crosslinking results in release of preformed mediators including histamine and tryptase, which then activate inflammatory cytokines and chemokines. It is this inflammatory cascade that leads to the symptoms of anaphylaxis.^2^

Although no tryptase level was confirmed in our case, the temporal relationship of symptoms to ketamine exposure point to significant histamine release and possible IgE-mediated anaphylaxis.^5^ However, an in-vitro study performed by Fell et al. demonstrated that ketamine directly increases histamine efflux in the brain, without mediation by IgE.^9^ One small study used the Prausnitz-Kutzner test to confirm that a ketamine allergic reaction was indeed not mediated by an IgE mechanism, and was more likely to result from direct ketamine stimulation of mast cells. Serum (suspected to contain IgE against ketamine) from the patient who had an allergic reaction to ketamine was placed intradermally (ID) in two healthy controls. Twenty-four hours later, different dilutions of ketamine were injected ID and observed. No reaction was noted, which provides evidence against an IgE-mediated allergic reaction.^6^

## CONCLUSION

Our case is consistent with a ketamine-induced adverse drug reaction, but whether ketamine directly stimulates mast cells to increase histamine release or causes an IgE-mediated anaphylactic reaction requires additional studies. The few studies available point to possible direct mast cell stimulation.^6^,^9^ Regardless, the end result can be quite similar and practitioners should be prepared to administer epinephrine, diphenhydramine, and steroids and be ready to establish an advanced airway. This is the only case of anaphylaxis to IM ketamine as monotherapy in the literature thus far; it serves as a reminder to be prepared for severe allergic reactions in the ED.
